# A Systematic Review on the Evolution of Power Analysis Practices in Psychological Research

**DOI:** 10.5334/pb.1318

**Published:** 2025-01-09

**Authors:** Lara Vankelecom, Ole Schacht, Nathan Laroy, Tom Loeys, Beatrijs Moerkerke

**Affiliations:** 1 Department of Data-Analysis, Ghent University, Belgium; 2 Department of Experimental Psychology, Ghent University, Belgium

**Keywords:** Replication crisis, statistical power, publication bias, systematic review, power analysis prevalence

## Abstract

Performing hypothesis tests with adequate statistical power is indispensable for psychological research. In response to several large-scale replication projects following the replication crisis, concerns about the root causes of this crisis – such as questionable research practices (QRPs) – have grown. While initial efforts primarily addressed the inflation of the type I error rate of research due to QRPs, recent attention has shifted to the adverse consequences of low statistical power. In this paper we first argue how underpowered studies, in combination with publication bias, contribute to a literature rife with false positive results and overestimated effect sizes. We then examine whether the prevalence of power analyses in psychological research has effectively increased over time in response to the increased awareness regarding these phenomena. To address this, we conducted a systematic review of 903 published empirical articles across four APA-disciplines, comparing 453 papers published in 2015–2016, with 450 papers from 2020–2021. Although the prevalence of power analysis across different domains in psychology has increased over time (from 9.5% to 30%), it remains insufficient overall. We conclude by discussing the implications of these findings and elaborating on some alternative methods to *a priori* power analysis that can help ensure sufficient statistical power.

## Introduction

Over the past decade, a series of events has cast doubt on the reliability of psychological research. Noteworthy among these were instances of research fraud committed by two social psychologists in 2011 and 2012 (Moussa & Charlton, 2023; Wicherts, 2011), as well as the publication of several controversial studies in the social and experimental psychology literature (e.g., Doyen et al., 2012; Wagenmakers et al., 2011). These events, among others, have led to a significant rise in publications addressing what is now often dubbed a replication crisis (e.g., Asendorpf et al., 2013; Colling & Szucs, 2021; Morawski, 2019; Pashler & Harris, 2012; Shrout & Rodgers, 2018; Stroebe & Strack, 2014; Świątkowski & Dompnier, 2017; Wiggins & Christopherson, 2019). The severity of the replication crisis became more pronounced when coordinated efforts to replicate many studies at once, such as the ‘Many Labs’ projects, were generally unable to do so convincingly (Ebersole et al., 2016; Ebersole et al., 2020; Klein et al., 2014; Klein et al., 2018; Klein et al., 2022; Open Science Collaboration, 2015). The rates with which these replications were successful ranged from a low 36% (Open Science Collaboration, 2015) to a somewhat more promising albeit still disappointing 54% (Klein et al., 2018).^1^ Furthermore, the effect sizes observed in these replication attempts were often considerably lower as compared to those in the original studies.

One common denominator in these replication projects was that they almost always represented a close replication (Stroebe & Strack, 2014). That is, the replication attempts made explicit efforts to deviate as little as possible from the original designs, oftentimes by contacting the original authors for specific information on the procedure. If we assume the replication procedure sufficiently precise, a failure to replicate is likely to point to either a lack of power in the replication design (Schauer and Hedges, 2020), or to the originally published effect being a false positive finding. Given that replication studies often employ larger sample sizes than the original studies to enhance statistical power, it is highly plausible that a high rate of false positive results in the original literature constitutes the primary reason for why replication attempts are often unsuccessful (Ioannidis, 2005; Szucs & Ioannidis, 2017). Although the false positive rate in psychological literature should be approximately equal to the α-level used in individual studies to make significance-based decisions (usually 5%), authors have argued that it is considerably higher due to the frequent use of questionable research practices (Simmons et al., 2011; Wicherts et al., 2016).

Questionable research practices (QRPs) have long been the primary focus in investigating the causes of the replication crisis. By introducing large amounts of false positive results into the literature, they render much of it largely irreplicable (in the sense of close replications). QRPs involve any action that deviates from established methodological standards and often fall into a gray area where the boundaries of acceptable conduct are not clearly defined (Linder & Farahbakhsh, 2020). QRPs come in a variety of flavors, but perhaps the most severe practice is p-hacking. P-hacking is an umbrella term for any *ad hoc* measure that a researcher applies to manipulate a previously non-significant p-value into a significant one (e.g., Friese & Frankenbach, 2020; Head et al., 2015). Subsumed under p-hacking are practices like *cherry picking* or *selective reporting* of statistical tests which have yielded significant p-values (i.e., p < .05), *optional stopping* or *data peeking* where statistical significance tests are iteratively conducted on accumulating data samples until a statistically significant result pops up and is subsequently reported on its own, and *selective data trimming* of results such that statistically significant results are obtained (see Stefan & Schönbrodt, 2023 for a detailed discussion of questionable p-hacking strategies). The practice of p-hacking allows for virtually anything to be presented as statistically significant, leading to a literature that is saturated with false positive results. P-hacking is a wide-spread phenomenon, as evidenced by survey results revealing that the percentage of researchers admitting to having employed a p-hacking strategy at least once is alarmingly high (Agnoli et al., 2017; Fraser et al., 2018; John et al., 2012; Swift et al., 2022).

It has been argued that the incentive for using p-hacking is mainly fueled by the fact that the current culture of publishing strongly favors significant results (Fanelli, 2012; Francis, 2012; Greenwald, 1975), a phenomenon known as publication bias. Combined with a historically evolved pressure to publish, this bias increases the motivation for researchers to resort to questionable research practices in a ‘final attempt’ to attain statistically significant and thus ‘publishable’ results (De Rond & Miller, 2005; Simmons et al., 2011). In recent survey research, respondents have freely admitted that one of the most common causes for self-assessed research misconduct is this ‘publish-or-perish’ academic culture (Pupovac et al., 2017). Consequently, calls for revisions of this biased, quantity-driven publishing culture have been and are still being made (Cumming, 2008; Nosek, Spies, & Motyl, 2012; Nosek & Lakens, 2014; Nosek et al., 2015; Rodgers & Shrout, 2018). For example, Nosek and Lakens (2014) introduced registered reports, where peer review is conducted based on pre-registered study proposals prior to data collection and analysis.^2^ Embracing registered reports as a publication standard modifies the incentive system for authors by reducing the pressure to pursue statistical significance, allowing them to focus more on the validity of the experimental procedures. Although evidence regarding improved research quality is currently still inconclusive, initial findings comparing registered reports to the standard publishing model are promising (Brodeur et al., 2022; Soderberg et al., 2021). As another example, Nosek et al. (2015) have formulated a series of author guidelines aimed at increasing transparency, openness, and reproducibility in empirical science (abbreviated as the TOP guidelines). This reform attempt describes four levels of transparency in different topics of academic publishing, ranging from data transparency to preregistration of analysis plans. The TOP guidelines have since been implemented by a range of APA journals.

So, literature on the causes of the replication crisis is vast and rich, and a sizable amount of it is focused exclusively on QRPs. However, this preoccupation has long drawn attention away from another practice that also perpetuates the replication crisis: a consistent failure to adequately consider statistical power in published research, both by individuals performing the research, as well as by their peers, whose role it is to evaluate and critically interpret a study’s findings. This negligence is worrying, given how low statistical power not only entails a low probability of finding a true effect, but can itself also yield an increased false positive rate in published literature (see the next section on Theoretical Background for details). In a seminal paper, Cohen (1962) already addressed the fact that statistical power in psychology should receive far greater attention from both individual researchers as well as from editors evaluating the work. His findings showed that most papers had a power of less than 50% for detecting small and medium effect sizes (see also Sedlmeier & Gigerenzer, 1989, for a follow-up 20 years later). More recently, Stanley, Carter, and Doucouliagos (2018) inspected 200 meta-analyses in psychological literature, spanning nearly 8000 individual empirical papers. Based on their findings, the authors concluded that the median power was only slightly above 36% and that only 8% of the studies were sufficiently powered to test their hypotheses.

One of the recurrent symptoms that illustrates this neglect for statistical power is the severely low reporting rate of power analyses in empirical articles. Several attempts have already been made to investigate the prevalence of power analyses in psychological research. For instance, Fritz, Scherndl, and Kühberger (2012) examined the reporting rate in different domains of psychology between 1990 and 2010. Drawing from meta-analytic data encompassing 1164 articles, they revealed that only 2.9% of the empirical studies reported a power analysis of some kind somewhere in the paper. In a similar vein, Tressoldi and Giofré (2015) found similar results based on 853 studies published in *Frontiers of Psychology* in 2014. Their analysis indicated that only 2.9% of empirical studies provided a justification for the sample sizes used, emphasizing the pervasive neglect of prospective power as a means for constructing strong experimental designs. As a final example, Vankov, Bowers, and Munafò (2014) analyzed 183 empirical studies published in *Psychological Science* in 2012. They report that only 6% of the studies made some mention of statistical power. The 183 original authors were subsequently contacted to establish the rationale used for deciding their sample size. Based on 94 returned responses, Vankov et al. (2014) concluded that formal power analysis was used in only 9.6% of the empirical studies.

Fortunately, in the last few years, the importance of statistical power has been increasingly recognized, for example, as more funding bodies and publishers explicitly demand formal power analyses to be conducted and reported (e.g., APA Journal Article Reporting Standards (JARS); see Appelbaum et al., 2018; Levitt et al., 2018). Equally promising is the steady increase in the publication of so-called power primers, the goal of which is to provide extensive instructions for researchers on how to perform power analyses in specific analytical circumstances (e.g., Brysbaert, 2019; Perugini et al., 2018; Wang, 2023). With all these recent efforts and the recent increased recognition of the possible role of low statistical power in sustaining the replication crisis (Stanley et al., 2018), the question arises whether power analysis prevalence, which was found to be dramatically low by earlier research, has now effectively gained prominence in the empirical literature. Recent research by Fraley et al. (2022) suggests as much. They found that the statistical power of studies was inadequate (with statistical power estimated around 50%) for six out of nine major social psychology journals in 2011, whereas only one of these journals remained inappropriately powered in 2019.

This article presents a systematic review examining the power analysis practices in 903 articles, published in 24 APA journals belonging to four distinct psychological disciplines. In this review, we compare articles published in the mid 2010’s when the replication crisis and its causes were a trending topic (i.e. 2015–2016) with articles published five years later (i.e. 2020–2021). Before discussing the set-up and results of the review, we will first elaborate on how underpowered studies, in combination with publication bias, contribute to a literature abundant with false positive results and overestimated effect sizes. A discussion of the notion of statistical power is necessary to fully understand its adverse impact on both the individual paper and the body of literature the paper pertains to. After discussing these theoretical grounds, we introduce the systematic review and descriptively report on the results of power analysis prevalence over time, across different psychological disciplines and different journal impact factor (JIF) quartiles. We make a comparison of the sample sizes of studies with and without a reported power analysis and investigate the power analysis practices in more detail for the former.

## Theoretical Background

The frequentist approach remains the gold standard for statistical inference in psychology. It involves translating a theoretical question into two competing hypotheses: the null hypothesis (H_0_), which typically assumes no effect, and the alternative hypothesis (H_1_), which assumes there is an effect. Data is collected and summarized using a test statistic, and we use the distribution of this test statistic under the assumption that H_0_ is true (called the null distribution) to measure evidence against H_0_ based on the data. This process leads to a decision: either reject H_0_ or fail to reject it. The decision process is designed so that if we were to repeat the study many times, we would make incorrect decisions about H_0_ at certain frequencies. More specifically, if H_0_ is true, we allow for a small probability (commonly set at .05; denoted by α) of wrongly rejecting it (also called a type I error). If H_1_ is true, we allow for a small probability (typically set at .10 or .20, denoted by β) of wrongly failing to reject H_0_ (also called a type II error). However, because the decision process only builds in a limit for the type I error rate α, the type II error rate β must be more purposefully controlled. Such strict control is achieved by adjusting the study’s design parameters – including the desired significance level (α), the sample size (N), and the effect size (ES) – to align with a test-specific power function. Statistical power, defined as the probability of detecting an effect if one exists (i.e. 1 – β), depends on these parameters (Cohen, 1992).

There are two main strategies to manipulate the design parameters, both of which make use of the fact that α, β, N and ES are interrelated for any given statistical test and study design. The first approach consists of *a priori* fixing both α and β to set acceptable limits for type I and type II errors, and determining a minimum meaningful effect size (sometimes dubbed the smallest effect size of interest, or SESOI, denoting the smallest ES that would be meaningful to uncover for the given research question), and then calculating the minimum sample size needed to achieve the desired power. This procedure is what is traditionally known as an *a priori* power analysis. However, practical restrictions on the available sample size or underdeveloped prior notions regarding the effect size of interest may render this first approach untenable in practice, in which case one may opt for a sensitivity analysis instead. Here, the sample size is fixed, and one assesses either the minimal ES detectable given the chosen α and β, or the attainable power given the chosen α and ES.

If statistical power turns out to be lower than desired (e.g. the collected sample size is smaller than required by the *a priori* power analysis, or the minimal detectable ES is greater than the prespecified SESOI), the result is a higher probability of false non-rejections of H_0_ (i.e. a larger type II error rate). At the level of individual studies, underpowered designs constitute a waste of resources in the form of money, energy, and time, due to the reduced ability of such studies to detect true effects. This problem is exacerbated by the current publication culture, where negative and null findings (whether true or false) tend not to get published (Rosenthal, 1979; Francis, 2012). For example, if one research group fails to find an effect due to low statistical power *and fails to report on it*, another may try anew and waste resources in a similar fashion out of ignorance. Apart from this more obvious source of waste, low statistical power may also be related to the degree of QRP adoption. If an experiment is underpowered (e.g., small SESOI combined with small sample size), a researcher might feel tempted to toy around with their analyses until the frequently misused benchmark for publication, namely statistical significance, is achieved (Ferguson & Brannick, 2012). This practice may maximize the individual researcher’s chances of publication, but leads to a biased, largely false-positive literature, hampering the desired cumulative nature of scientific knowledge.

**Figure 1:**
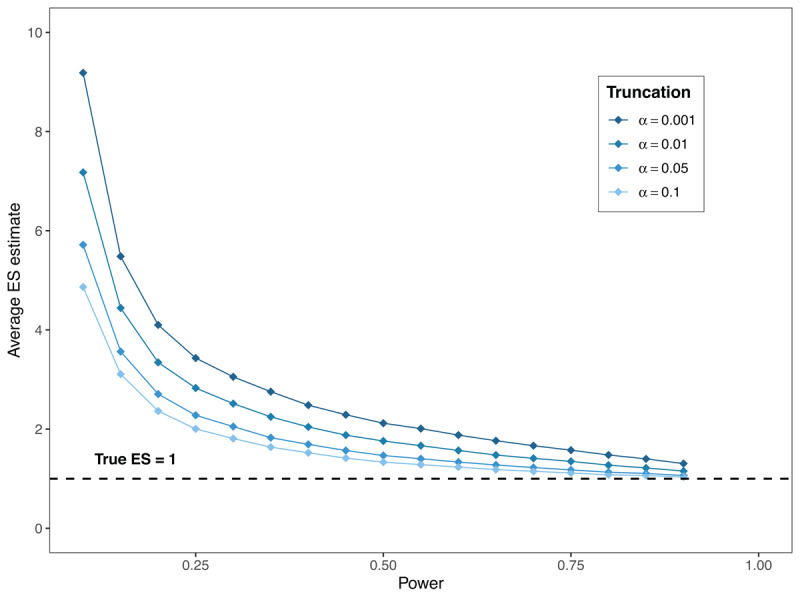
The amount of effect size overestimation after truncation for different statistical power levels (for the Student t-test).

Another common issue with underpowered research in combination with the current publication culture is that it often results in overestimated effect sizes (Ioannidis, 2008). To understand why low power leads to overestimated ES, consider the following reasoning. For a low-powered study to yield a statistically significant and thus ‘publishable’ result (without the use of QRPs), the gathered data in the sample must display extreme values in favor of the alternative hypothesis. This causes smaller true effect sizes to appear larger in the sample than they are in the population. This implies that, on average, the ES estimates contained within an underpowered and biased body of literature (where only statistically significant results are retained) paint a more extreme picture of reality than is warranted (Ioannidis, 2005; Stanley, Doucouliagos, & Ioannidis, 2021). This phenomenon is also known as the winner’s curse (Lindstromberg, 2023; Van Zwet & Cator, 2021). As shown in Figure 1, truncating results based on statistical significance (i.e. keeping only significant findings) biases effect size estimates upward, and this bias worsens as statistical power decreases. Stricter thresholds for significance (e.g. p < .01 or p < .001) further exacerbate these overestimations, as only the most extreme data are retained. This bias can be mitigated by also including non-significant findings in the literature. The supplementary materials (available on the OSF-repository) provide some further background and reflection on the winner’s curse for the interested reader.

A final issue, equally problematic, concerns the predictive accuracy of statistical evidence (Button et al., 2013). Traditional statistical decision-making relies on probability measures that are conditional on specific assumptions – namely, whether the null hypothesis being tested is true. As already mentioned earlier, α defines the probability of rejecting H_0_ when it holds, and β represents the probability of not rejecting H_0_ when it does not hold. These probabilities are central to frequentist decision theory, as they serve to guide decision-making about hypotheses in a structured manner. Yet, interest often lies in the reversed probabilities, namely, the probability that H_0_ is false given that one has rejected it, and the probability that H_0_ is true given that one has failed to reject it. Known as the positive predictive value (PPV) and negative predictive value (NPV), respectively, these values represent the proportion of correct rejections and correct non-rejections out of all rejections and non-rejections. High PPV and NPV are desirable, as they indicate that the majority of statistical decisions are accurate – i.e., that significant results reflect true effects (true positives), and non-significant results correctly indicate the absence of an effect (true negatives). The PPV and NPV in a body of literature depend on the significance level, the statistical power of studies, and on the prior probability of H_0_. As statistical power in the research field increases, both PPV and NPV improve; however, this improvement is more pronounced for PPV when the prior probability of H_0_ is high, and for NPV when the prior probability of H_0_ is low. For a more in-depth discussion on the interplay between power, significance level, and prior likelihood of H_0_ on the PPV and NPV; see supplementary materials.

In conclusion, low-powered studies do not only contribute to a literature rife with false-positive results and overestimated effect sizes, they also reduce both the positive and negative predictive value of findings. Fortunately, awareness of the critical role of statistical power in ensuring the credibility of empirical research is growing (e.g., Bishop, 2019; Ioannidis, Stanley, & Doucouliagos, 2017). Publishers now often advocate for sample size calculations, and a growing number of accessible power primers are emerging with the aim to guide applied researchers in conducting statistical power analyses. Consequently, there may be reason to suspect that power analysis is gaining increased attention, including in how frequently it is reported in empirical research. To investigate this conjecture, we present the results of a systematic review based on 903 empirical articles published in psychology.

## Systematic Review

### Procedure

To assess how reporting of power analysis and practices therein have evolved over time, we conducted a systematic review based on 903 empirical articles published within the domain of psychology. The scope of this systematic review was constrained to journals available through the *American Psychological Association* (APA). In particular, interest was in comparing the following four APA-disciplines: (i) clinical psychology, (ii) experimental psychology, (iii) educational psychology, and (iv) social psychology. Detailed information on these four APA-disciplines and their corresponding journals is available on the APA-discipline homepage.^3^ Six journals were randomly sampled within each of the four selected APA-disciplines. An APA-journal was eligible for selection when (i) it had a journal impact factor (JIF), (ii) it was listed on the Web of Science core collection database, and (iii) its APA-discipline listing did not conflict with the scientific discipline mentioned on Web of Science. When selecting journals, we made a concerted effort to include journals from all four JIF quartiles.

The main goal of the systematic review was to identify if and how the prevalence of power analysis has evolved since the publication of several important papers addressing the replication crisis around the year 2015. To assess this trend, we investigated power analysis practices in two time periods five years apart (2015–2016 versus 2020–2021). For three out of the six journals within each APA-discipline, we randomly analyzed 20 articles in 2015 and 20 articles five years later in 2020. The remaining three selected journals within each APA-discipline were analyzed in the exact same way but the years of publication were 2016 and 2021. The choice for which journals to analyze in 2015 (and 2020) versus 2016 (and 2021) was purely coincidental, and driven by multiple master thesis projects performed by graduates starting in different academic years at the faculty of psychology and educational sciences of Ghent University (Belgium). This design enabled us to compare the prevalence of power analysis within each journal over a five-year period, as well as to find more general trends over time, discipline, and JIF quartile.

To be eligible for inclusion, a research article had to conduct a quantitative primary analysis based on empirical data that was not published previously (so no secondary analyses, reviews, or meta-analyses) and had to be written in English. The selection procedure resulted in (at best) 40 empirical articles within each of the selected journals (20 articles per year of publication). Unfortunately, some journals did not contain 20 empirical articles within a given year of publication which hampered our initial goal of 960 articles. Table 1 shows the number of articles within each journal and year that were included in the systematic review. In total, 903 empirical psychology articles were analyzed for power analysis practices. The disciplines of social, experimental, and clinical psychology have nearly complete data. For two journals within the educational discipline, not enough empirical articles met the inclusion criteria.

**Table 1:** Overview of included journals for the four APA-disciplines and the number of articles analyzed within each journal and year. The JIF quartiles of each journal are displayed between brackets for the time periods 2015–2016 (first position) and 2020–2021 (second position).


DISCIPLINE	JOURNAL	YEAR OF PUBLICATION			

		2015	2016	2020	2021

Clinical	Psychological Trauma Theory Research Practice and Policy *(Q2 – Q2)*	20		20	

	Rehabilitation Psychology *(Q3 – Q3)*	20		20	

	Health Psychology *(Q1 – Q1)*	20		20	

	Journal of Consulting and Clinical Psychology *(Q1 – Q1)*		20		20

	Journal of Experimental and Clinical Psychopharmacology *(Q2 – Q2)*		20		20

	Psychological Services *(Q3 – Q3)*		20		20

Experimental	Journal of Experimental Psychology: General *(Q1 – Q1)*	20		20	

	Journal of Comparative Psychology *(Q2 – Q3)*	20		20	

	Journal of Experimental Psychology: Human Perception and Performance *(Q2 – Q2)*	20		20	

	Emotion *(Q1 – Q1)*		20		20

	Journal of Experimental Psychology: Learning, Memory, and Cognition *(Q2 – Q2)*		20		20

	Canadian Journal of Experimental Psychology *(Q4 – Q4)*		20		20

Social	Journal of Personality and Social Psychology *(Q1 – Q1)*	20		20	

	Group Dynamics: Theory, Research, and Practice *(Q4 – Q4)*	16		13	

	Law and Human Behavior *(Q1 – Q2)*	20		20	

	American Journal of Orthopsychiatry *(Q1 – Q1)*		20		20

	Cultural Diversity & Ethnic Minority Psychology *(Q2 – Q2)*		20		20

	Psychology of Men & Masculinities *(Q2 – Q3)*		20		20

Educational	Journal of Educational Psychology *(Q1 – Q1)*	20		20	

	Training and Education in Professional Psychology *(Q3 – Q4)*	13		9	

	Journal of Diversity in Higher Education *(Q4 – Q2)*	4		8	

	Journal of Counseling Psychology *(Q1 – Q1)*		20		20

	School Psychology (Quarterly) *(Q1 – Q2)*		20		20

	Canadian Journal of Behavioural Science *(Q4 – Q1)*		20		20


From each selected article, the following information was collected: (i) article meta-data (title, authors, keywords, etc.), (ii) the sample size(s) used in the study (before and after exclusion criteria), (iii) the presence of a power analysis, and if so, (iv) whether the power analysis was performed as an *a priori* power analysis, *post hoc* power analysis, or sensitivity analysis,^4^ (v) the desired/obtained power of the study and the α-level used in the power analysis, (vi) whether the power analysis was based on a standardized or unstandardized effect size, (vii) the type and the magnitude of the effect size used in the power analysis, and finally (viii) the way in which the relevant effect size was obtained (literature, pilot data, rule of thumb, etc.).

A number of included articles consisted of multiple studies or experiments, each using a different sample of participants. This phenomenon was particularly notable among Q1 journals within the APA-disciplines of experimental and social psychology. Overall, 36.4% of all included articles contained at least two studies or experiments with different participant sets. To prevent these articles from disproportionately influencing the results, we restricted each article to just one study or experiment. The process of selecting one study or experiment per article was carried out after information on all experiments was collected. When none of the studies or experiments performed a power analysis, we retained the one that had the highest sample size after exclusion. When at least one of the studies or experiments contained a power analysis, we selected the one with the power analysis, where *a priori* was preferred over sensitivity analysis and sensitivity analysis was in turn preferred over *post hoc*. When more than one study or experiment remained, the one with the highest sample size after exclusion was again chosen. Filtering the data set according to this decision rule ensured that the results represent the most optimistic case.

The data collection was carried out by all authors and several graduate students under supervision of the first author, as part of their dissertation. Questionable or vague cases were discussed in separate meetings to increase the inter-rater reliability. All articles for which a power analysis was conducted were checked afterwards by the main author to ensure correctness and uniformity. After preparing the dataset, we used R for further analysis. All material to reproduce the results reported below is available on the OSF-repository.

## Results

### Power analysis prevalence

**Figure 2:**
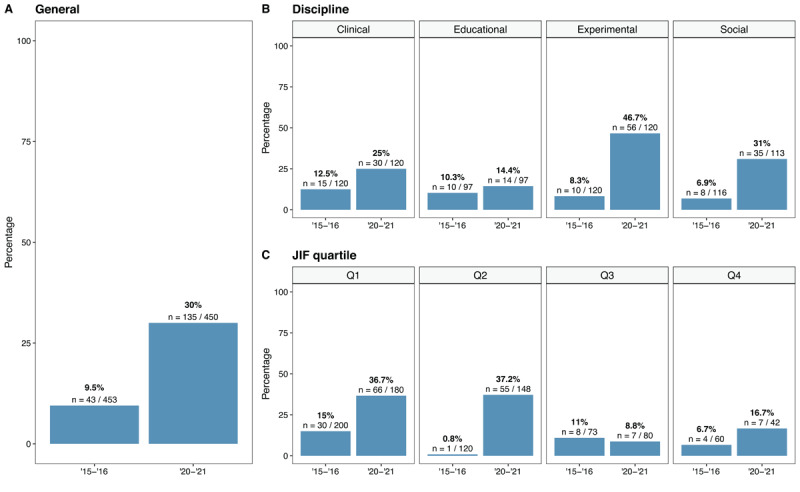
Evolution of power analysis prevalence in general (panel **A**), per discipline (panel **B**), and per JIF quartile (panel **C**).

Our main research question concerned the evolution of prevalence with which power analysis was conducted in 2015–2016 versus 2020–2021. Figure 2 shows the percentage of articles with a power analysis over time for three levels: overall prevalence (panel A), discipline-specific prevalence (panel B), and JIF quartile-specific prevalence (panel C). A promising evolution in the frequency of power analysis can be observed across all three levels. Specifically, the aggregated results show that the prevalence of power analysis has increased from 9.5% in 2015–2016 to 30% in 2020–2021. Discipline-specific and JIF quartile-specific percentages yield more refined information. While we observe that the prevalence of power analysis has increased across all considered APA-disciplines (however, only a negligible increase in educational psychology), the disciplines of experimental psychology and social psychology have made the strongest improvement (from 8.3% to 46.7% and from 6.9% to 31%, respectively). Concerning the JIF quartiles, Q1 and Q2 journals have made more progress over the span of five years as compared to Q3 and Q4 journals.^5^ The highest quartile-specific increase is noted for articles published in Q2 journals (from 0.8% to 37.2%).

**Figure 3:**
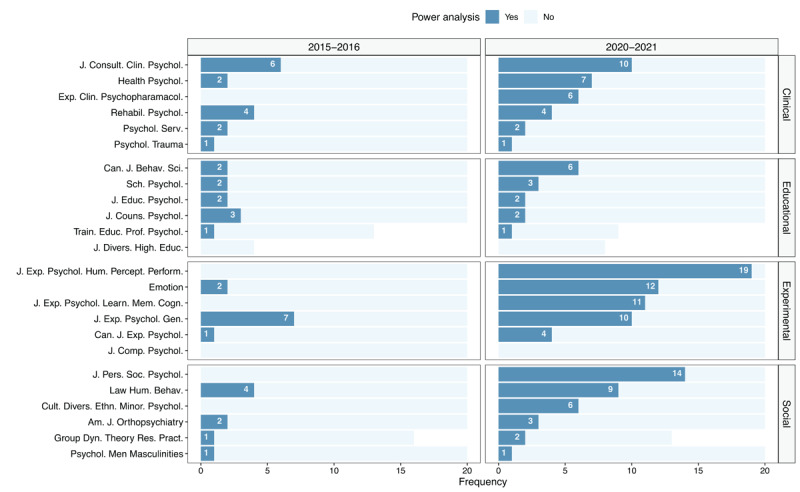
Number of articles with and without a power analysis across the two time periods within each of the included journals.

Figure 3 shows the evolution of power analysis prevalence for each journal separately. The practice of reporting a power analysis has increased (or remained the same) in all journals as compared to the first time period (with the exception of a negligible decline in the *Journal of Counseling Psychology* from 3 articles in 2016 to 2 articles in 2021). Several journals show a notably large increase. Upon further research we note that some of these journals have strongly encouraged or even introduced binding policies on power analysis and sample size justification after the year 2016. As an example, the *Journal of Experimental Psychology: Human Perception and Performance* made a remarkable increase in studies that explicitly mention power analysis, starting from zero of the 20 sampled articles in 2015 to 19 out of the 20 articles in 2020. Illustrative of this sharp increase is the following editorial, published in 2018:

*‘[…] decisions about sample size have historically not been well motivated in Psychology, and we now request more explicit information about such decisions. If you choose to use frequentist inference, we will ask you to make an explicit case that you have sufficient a priori power or that special conditions may justify an exception. The Journal welcomes […] Bayesian analysis, but in all cases authors need to be transparent about their method including how their sample size was chosen and make a case that it is sufficient, given the study’s objectives.’* (Gauthier, 2018, p. 1).

Aside from the editorial, a strong justification for sample size is also explicitly mentioned in the submission guidelines of the journal. In line with the TOP guidelines introduced by Nosek et al. (2015), authors are required to include a subsection titled ‘transparency and openness’, where the rules for sample size determination should be detailed. The journal also mandates the use of APA Journal Article Reporting Standards (JARS) throughout the manuscript (Appelbaum et al., 2018).

A second example concerns the *Journal of Personality and Social Psychology*, where power analysis prevalence has increased from zero studies in 2015 to 14 of the randomly sampled articles in 2020. The journal also makes explicit references to the JARS, as well as the specific TOP guidelines that should be adhered to. In their 2017 editorial, explicit emphasis is placed on transparency and sample size justification:

*‘First, we must place a greater emphasis on the robust demonstration of a key effect in a given project. This will require the use of an adequately powered design, which often requires larger sample sizes. […] [T]he authors should provide a clear, carefully crafted rationale(s) for the target N for each study. It bears emphasis that certain statistical formalities including power analysis may be part of this rationale and, in fact, we encourage the use of these tools whenever doing so is reasonable. […] Authors must provide a broad discussion on how they sought to maximize power. This discussion should include, but not be limited, to sample size.’* (Kitayama, 2017, p. 357–358).

We further note that all journals for which a large increase in power analysis prevalence is observed include sample size justification as an important tenet in their editorial notes and submission guidelines. For most journals, these regulatory efforts had a positive effect on the prevalence of power analysis, although this is not a guarantee for success. For instance, in their 2018 editorial (Ehde, 2018), *Rehabilitation Psychology* states that the APA JARS (Appelbaum et al., 2018) should be adhered to when submitting a manuscript. The submission guidelines also mention that authors should include a section on transparency and openness that discusses, among other things, sample size determination. In the current systematic review however, the relative number of power analyses in this journal remained constant and low overall despite these policy changes. A similar observation can be made for the *Journal of Educational Psychology*.

### Power analysis practices

**Figure 4:**
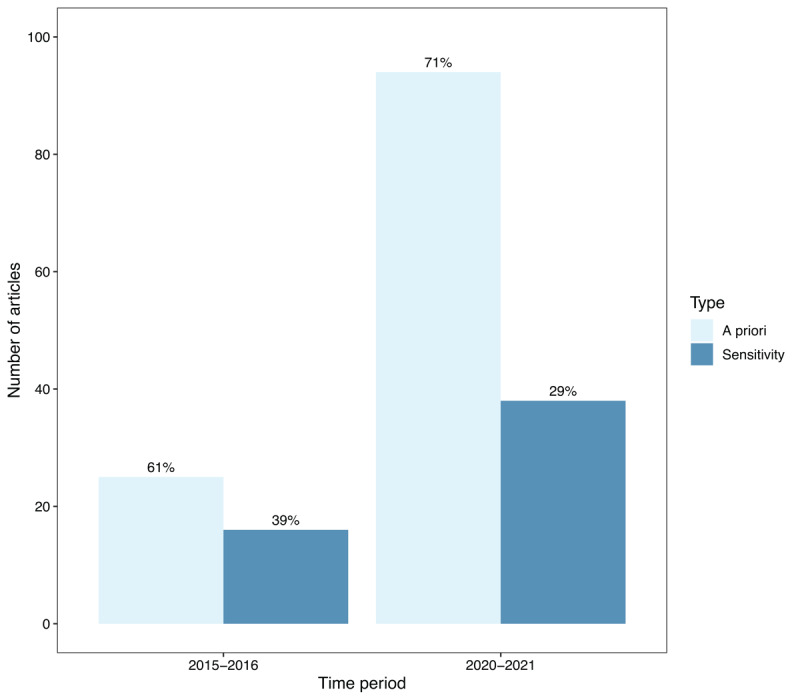
Ratio of *a priori* power analysis versus sensitivity analysis over time.

In addition to the evolution in power analysis prevalence, we also investigated the common practices when power analysis was effectively reported. The analyses in this section therefore only consider the 178 empirical articles (19.7%) that described a power analysis. Out of these 178 articles, only five articles performed a *post hoc* power analysis (2 articles in 2015–2016 and 3 articles in 2020–2021). The remaining 173 articles performed either an *a priori* power analysis or a sensitivity analysis. Figure 4 shows the number of articles that reported an *a priori* power analysis and a sensitivity analysis across the two time periods. Once again, a promising evolution can be detected. Apart from the considerable surge in the number of articles incorporating a power analysis during 2020–2021 compared to 2015–2016, there has also been a noteworthy increase in the proportion of *a priori* power analyses (relative to sensitivity analyses) from 61% in 2015–2016 to 71% in 2020–2021.

For the 173 empirical articles that reported an *a priori* power analysis or sensitivity analysis, we investigated three essential ingredients: power, α-level, and effect size. In line with common benchmarks, the majority of the power analyses aimed at reaching a power of 80% (62.7% of studies), 13.4% of the studies a power of 95%, 6.7% a 85% power, 6.7% a 90% power, and 2.2% a 99% power.^6^ In 8.2% of the articles, no concrete information was provided on the desired power level, and terms like ‘sufficiently powered’ or ‘decently powered’ were used instead. There is no noticeable shift in the desired power levels used in 2015–2016 and those in 2020–2021. The significance level α used in the power analysis is the element that is most often omitted in the description. In 49.1% of the articles, this information was not explicitly provided. The remaining articles mainly reported using an α of .05 (45.1%), followed by an α of .01 (3.5%), an α of .025 (1.7%), and an α of .001 (0.6%).

A third essential element for performing a power analysis is an effect size. From the 173 studies that reported a power analysis, 47.5% derived their effect size from the available literature, 35.4% constructed the effect size based on a rule of thumb, and 5.1% conducted a pilot study prior to data collection to obtain an estimate.^7^ In 12% of the articles, the way in which the effect size was obtained was not discussed. The practice of defining the effect size based on a rule of thumb has decreased from 2015–2016 (41%) to 2020–2021 (33.6%), while the practice of using pilot data to estimate the effect has increased from 0% to 6.7%. Of all studies performing a pilot study, only one did not provide any information on the executed pilot. Looking at historical data to define the effect size seems to remain the most used strategy both in 2015–2016 (46.2%) as well as in 2020–2021 (47.9%).

**Figure 5:**
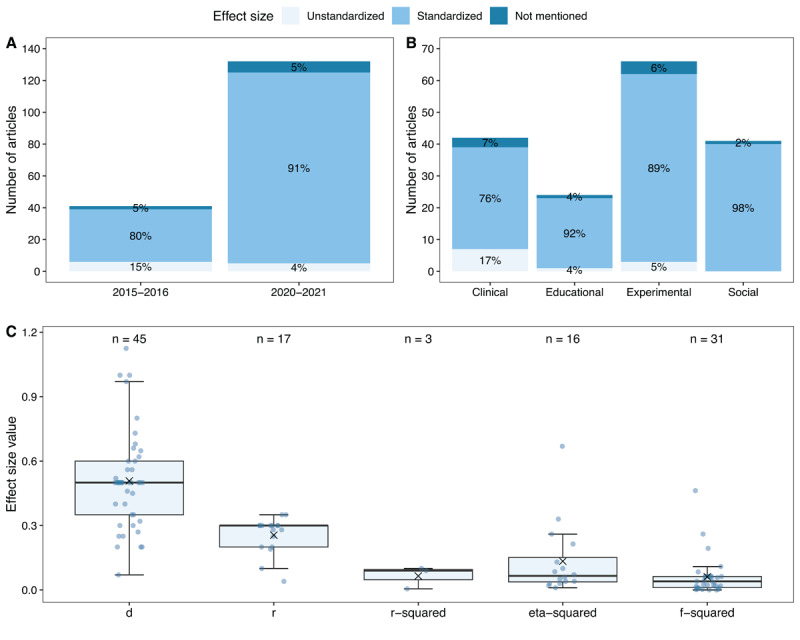
Standardized versus unstandardized effect sizes used in power analysis. *Note*. Panel A and B are based on the 173 studies that report an *a priori* power analysis or sensitivity analysis. Panel C is based on the 112 articles that employed (and reported the type of) standardized effect in the power analysis and shows the values used for each type of standardized effect.^8^ The mean of the distribution is indicated by the cross.

When delineating the effect size for power analysis, researchers may opt to specify it either in unstandardized or standardized terms. Figure 5 (panel A) shows that the proportion of articles using an unstandardized effect in the power analysis is low in 2015–2016 (15%) and even lower in 2020–2021 (4%). While the call to conduct more power analyses has clearly reached the applied researcher, it appears that the routine of using unstandardized effect sizes instead of standardized ones has not yet become prevalent, despite its potential benefits in gaining greater control over the power of the study (Baguley, 2009). In both time periods, 5% of the articles did not contain any information on the used effect size. Figure 5 (panel B) illustrates these rates per discipline (aggregated across both time periods). While in every discipline, the majority of the articles employed standardized effect sizes, the clinical discipline used remarkably more unstandardized effects in their power analyses compared to the other disciplines (17% compared to 4%, 5%, and 0% for educational, experimental, and social psychology, respectively). A plausible explanation for this trend might be that the discipline of clinical psychology is more closely related to the field of clinical trials, where the practice of performing power analysis with unstandardized effect sizes is more natural (Davis et al., 2020). For the articles using standardized effect sizes in their power analysis (n = 153), we looked into the type and value of the employed standardized effect (Figure 5, panel C). Cohen’s d is the effect size that was most often reported (n = 45), followed by f-squared (n = 31), correlation *r* (n = 17), (partial) η-squared (n = 16), and R-squared (n = 3). Remarkably, the median value for all these effect size types in our sample corresponds to the value of a medium effect size according to Cohen’s rules of thumb (1988). A considerable number of papers failed to specify the type of effect size used in the power analysis (n = 38). Among them, 22 articles solely presented the numerical value used as effect size without additional information, while 16 articles did not offer a specific numeric value, opting instead for vague terms such as ‘medium’ or ‘small-to-medium’ effect. Finally, while only 46.2% of the 173 articles allow for full reproducibility through reporting their power analysis in full detail, more than half of the articles remain vague on one or more of the input parameters.

### Sample size comparison

**Figure 6:**
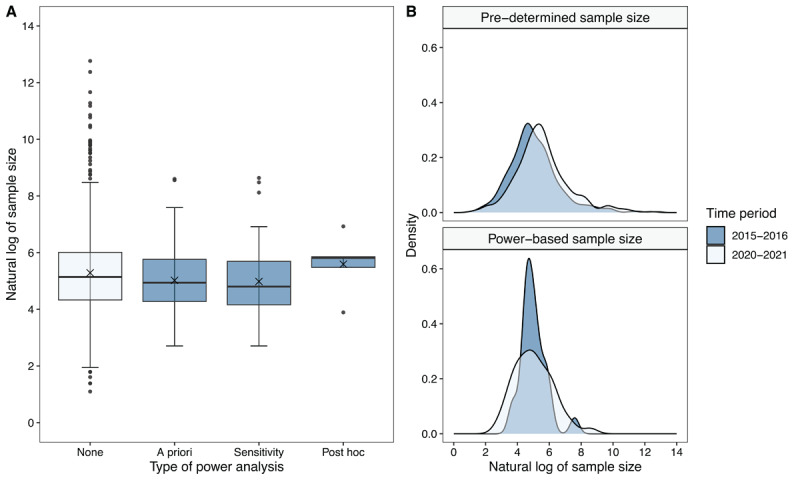
Distribution of the (log-transformed) sample sizes **A**) per type of power analysis, aggregated across time periods, disciplines, and JIF quartiles (*no power analysis*, n = 725; *a priori power analysis*, n = 119;, *sensitivity analysis*, n = 54; *post hoc power analysis*, n = 5), and **B**) for articles where the sample size was either pre-determined (2015–2016, n = 428; 2020–2021, n = 356) or power analysis-based (2015–2016, n = 25; 2020–2021, n = 94) per time period.

Finally, we present the results on the distribution of sample sizes for all included studies in the systematic review. Articles were grouped with respect to the type of power analysis that was reported. Figure 6 panel A shows the boxplots of the sample size distributions, aggregated across time periods, disciplines and JIF quartiles. The logarithmic transformation was used to display the sample sizes due to extreme right skewness impeding visual analysis. As shown in the boxplots, empirical articles in our review that did report a power analysis (*a priori, post hoc*, or sensitivity analysis) have considerably less variability in their sample sizes as compared to studies that did not include a power analysis. For this latter category (80.3% of articles), we observe a large dispersion in sample sizes, with studies ranging from 3 to 350.000 observations. In contrast, the median as a robust measure of central location seems to be relatively similar across all groups.

Furthermore, investigating whether sample sizes have increased in 2020–2021 compared to 2015–2016 may yield valuable insights. We categorized the articles into two groups: those that did not determine their sample size based on a power analysis (including articles with no power analysis or a sensitivity/*post hoc* power analysis), and those that utilized an *a priori* power analysis to decide on their sample size. Figure 6 Panel B illustrates the density of the log-transformed sample sizes for each of these categories across the two time periods. Although the shape of the distribution remains consistent over time, a noticeable rightward shift (toward larger sample sizes) is evident in 2020–2021 for studies where the final sample size was not determined based on a power analysis, relative to 2015–2016. This suggests that sample sizes are generally larger now, even among studies without power analysis-guided sample sizes. In contrast, for studies that used an *a priori* power analysis, the average sample size does not appear to have increased significantly; however, there is greater variability in final sample sizes in 2020–2021 compared to 2015–2016.

As Figure 6 shows, the average sample size in all selected articles is approximately 150 (obtained after exponentiating the log-transformed value for the average sample size). We performed sensitivity analyses to examine what the minimum detectable effect size is with this sample size to reach a power of 80% for the following common experimental designs (Brysbaert, 2019): 1) paired samples t-test, 2) independent samples t-test, 3) 2 × 2 repeated-measures ANOVA (i.e. a full-factorial design with two within factors), and 4) 2 × 2 split-plot ANOVA (i.e. a full-factorial design with one within and one between factor). Specifically, in the latter two designs, we considered two factors: factor 1 and factor 2. In design 3, both factor 1 and factor 2 are within-subjects factors, whereas in design 4, factor 1 is a within-subjects factor, and factor 2 is a between-subjects factor. We focused on testing the interaction effect for settings where factor 1 has an effect at one level of factor 2 and no effect at the other level of factor 2. To standardize the effect sizes across the designs, we used d_av_, a variant of Cohen’s *d* that is designed to be comparable across both within-subjects and between-subjects designs (Brysbaert, 2019; Lakens, 2013). This metric incorporates the correlation between repeated measures in within-subjects designs, while also standardizing the effect size based on the pooled variability across conditions. By using d_av_, we ensure that the minimum detectable effect sizes are directly comparable across all four experimental settings. We relied on the simulation code provided by Brysbaert (2019) to mimic data from these four designs, and to derive minimal detectable effect sizes (see Table 2; code is available on the OSF-repository). Following Cumming’s (2012) suggestion that d_av_ can be interpreted using the same benchmarks as Cohen’s *d*, the results show that a sample size of 150 participants can uncover small-to-medium effect sizes across all four experimental settings. Studies with a within-subject design (especially when the correlation between the repeated measures is high) can detect smaller effect sizes with 80% power than studies with a (partially) between-subject design. These results are in line with what was already described by Brysbaert (2019).

**Table 2:** Overview of the minimal detectable effect size d_av_ with a total sample size of 150 participants (ppt) to reach a power of 80% for different values of correlation between the repeated measures (not applicable for the independent samples t-test). Results are based on 5000 simulations.


SETTING	N	CORRELATION		

		r = 0.3	r = 0.5	r = 0.7

Paired t-test	Same 150 ppt in both conditions	0.27	0.23	0.18

Independent t-test	Different 75 ppt per group	0.46		

ANOVA 2 × 2 rep. meas.	Same 150 ppt in all 4 conditions	0.39	0.33	0.26

ANOVA 2 × 2 split-plot	Different 75 ppt per group	0.54	0.46	0.36


## Discussion

The past fifteen years have proven to be turbulent for psychological science. The large number of publications demonstrating the limited replicability of psychological effects exemplifies this notion (e.g., the ManyLabs projects; Open Science Collaboration, 2015), together with the increasing awareness regarding the widespread adoption of QRPs (John et al., 2012). Both the credibility (Pashler & Wagenmakers, 2012) and the validity of our field at large (Giner-Sorolla, 2018) have been called into question. Recurring arguments as to why empirical results often fail to be replicated point out that the empirical literature is rife with false positives, due to the exorbitant adoption of QRPs such as p-hacking, which is sustained and incentivized by a publication culture that emphasizes ‘unexpected’ or ‘new’ findings in the form of statistically significant results over rigorous scientific work that may be less exciting (Ferguson & Brannick, 2012). As a result, recent scientific reform efforts have primarily concentrated on corrective measures aimed at reducing researchers’ degrees of freedom, e.g., preregistration (Nosek et al., 2018), and at addressing the problematic aspects of the publish-or-perish culture, e.g., registered reports (Nosek & Lakens, 2014).

In this paper, we have drawn attention to another element of this picture which has historically remained neglected by the applied researcher: statistical power. For decades, systematic reviews have brought to light the fact that published psychological research is generally underpowered (Cohen, 1962; Fraley & Vazire, 2014; Maxwell, 2004; Sedlmeier & Gigerenzer, 1989; Stanley et al., 2018). Here, we have discussed two expected consequences: effect size overestimation due to publication bias and low positive predictive value. The aforementioned emphasis on statistical significance as a proxy for scientific evidence leads to the introduction of systematic bias in the magnitude of published effect sizes, painting a distorted picture of reality that hampers cumulative science. It also helps to explain why replicability (in the close sense) is so poor overall, and why replicated effect sizes tend to be smaller than those originally published. The low positive predictive value that accompanies low power is problematic considering rampant publication bias toward confirmatory findings (Francis, 2012).

Even though the reform movements’ preoccupation with false positives seems to have largely ignored statistical power as a potential culprit in this crisis, the topic has fortunately been gaining attention in recent years (Fraley et al., 2022; Munafò et al., 2017). Publishers nowadays often promote to include statistical power analysis in manuscripts, and the literature has seen an increase in layperson-friendly primers guiding applied researchers through the specifics of statistical power analysis and sample size justification (e.g., Lakens, 2022). The question arises whether this trend is mirrored by an increase in the reporting prevalence of statistical power analyses in empirical publications. To assess this, the current paper investigated the evolution of power analysis reporting prevalence across four prominent APA-disciplines.

The primary finding of the current systematic review is that we observed a sizable increase in the reporting prevalence of power analyses in empirical publications, from less than 10% to 30% across all journals considered. The greatest improvement is seen in the experimental and social disciplines, with the former having its proportion of publications including some form of power analysis more than quintupled, up to 46%. In terms of journal impact factor quartiles, the greatest improvement occurred in the investigated Q1 and Q2 journals. At face value, these numbers are very promising, but to credit a potential increase in awareness at the level of individual researchers with such growth may be premature. For instance, we acknowledge that the highest journal-specific improvements can also be due to the introduction of regulatory policies making sample size justification mandatory, as some editorials we have quoted earlier suggest, and not so much the individual awareness itself. However, these apparent changes are therefore no less valuable, and they seem to converge with other recent evidence supporting the notion that statistical power is increasing, on average, in parts of psychology research (Fraley et al., 2022).

The included journals from clinical and educational psychology also indicate an increase in reporting prevalence, but to a far smaller degree. One possible reason is that these subfields – i.e., insofar as the journals under investigation reflect their respective subfields well – are simply slower to accept these required changes, or more stubborn in their ways. However, it might also simply be the case that power analyses as such are not as easily implemented for the subject matter these journals concern themselves with. Journals and/or whole domains may prefer different ways to justify their sample sizes for practical reasons. For instance, we found that forensically oriented journals in psychology (e.g., *Law and Human Behavior*) tended more to draw samples and data from pre-existing longitudinal databases, obtained through legal institutions or private health organizations. In such cases where data have already been collected, but are randomly subsampled, conducting an *a priori* sample size calculation may not be as applicable. Moreover, in the case of clinical psychology, specific clinical populations may not exist or be approachable in large enough numbers to be able to adhere to the outcome of *a priori* power analyses. However, it would still be relevant in those cases to conduct sensitivity analyses to evaluate the extent to which fixed sample sizes, study design, and analysis methods are able to detect practically relevant effect sizes for a given power level. The same holds true for educational psychology, where experimental design may be less flexible due to the non-manipulability of the environment the domain is concerned with, and so must be rigorously examined for sensitivity. Especially in those circumstances where research leads to practical policy, it is important to understand how part of the literature that is occupied with the same question is configured in terms of power and sensitivity and to be able to evaluate the quality of published evidence in terms of the effect size estimates or its predictive value overall. In the current review, sensitivity analyses were included under the umbrella term of ‘power analysis’, and so it remains worrying that the investigated journals coming from applied fields such as clinical and educational psychology seem to progress so slowly. Note that we explicitly refrain from drawing general conclusions about respective subfields, since the purpose of the current systematic review is merely descriptive and not inferential. Targeted research that is appropriately designed to answer inferential questions of the kind alluded to above, is required before we may draw firm subfield-wide conclusions.

The increase in reporting prevalence is good in terms of quantity. However, power analyses must be held to a qualitative standard as well in order for a quantitative increase to be meaningful and indicative of true progress. We draw attention to several elements. First, the current review shows that an overwhelming majority of the reported power analyses were *a priori* and not *post hoc*. This is positive since *post hoc* power analyses are problematic. When researchers calculate statistical power using the sample size and *obtained* effect size, this ‘observed power’ is isomorphic to the observed *p*-value (Hoenig & Heisey, 2001; Pek et al., 2024), and, as such, adds no new information. The fact that this kind of analysis was rarely reported is therefore encouraging. Second, there is a clear need for standardization with respect to how statistical power analyses are reported. Ideally, readers should be able to reproduce a power analysis using the information provided by an author, which at minimum ought to include the desired type I and II error rates, the employed effect size, and the statistical test it was employed for. In many cases, unfortunately, desired power was denoted not numerically, but verbally, e.g., ‘sufficient’ or ‘decent’, without further qualification. More problematically, the effect size was also often denoted verbally, e.g., ‘medium’ or ‘small-to-medium’, making it impossible to gauge as a reader what the result of the reported power analysis entails with respect to the measured data and the ensuing hypothesis test. Oftentimes, the employed software was omitted from the text as well, and in a large number of cases it was not clear which hypothesis test the power analysis was applied to when multiple tests were conducted. These omissions and ambiguities hamper reproducibility, peer assessment of experimental work and interpretation of statistics by third parties and should be avoided whenever possible.

Third, choices regarding desired power and effect sizes are often not substantiated. Desired power is typically set at 80%. It is unfortunate, though understandable, that simple benchmarks are implicitly or explicitly propagated throughout statistical, methodological, and applied literature. Type II errors are typically considered less important in general, and the probability of a type II error is therefore traditionally set at .20, contrary to type I errors for which the probability is mostly set at .05. This adherence to tradition is undesirable because it avoids the need for justified testing. Recently, Lakens et al. (2018) have argued that significance levels should be justified and may deviate from the conventional .05. For example, in animal research, where ethical considerations often require smaller sample sizes, the risk of false positives due to lower statistical power must be carefully weighed. Similarly, desired power should be justified in terms of how the content of an experiment at hand weighs against the prospect of a false negative decision in a statistical procedure, a potential effect size overestimation, or the endangerment of a literature’s predictive value. Researchers may also wish to balance type I and II error risks differently based on context, according to which one is deemed more problematic. However, many researchers may find it challenging to provide such justifications. To assist with this, we refer the reader to an extensive and detailed paper by Giner-Sorolla et al. (2024), wherein numerous aspects pertaining to power level justification, sample size justification, SESOI determination, etc., are discussed at length. Relatedly, a substantial portion of effect size values reported in the current sample are standardized, e.g., ‘d = 0.4’, attributed to work by Cohen (1962; 1988). However, Cohen (1962) devised such standardized benchmarks because he aimed to address power ‘for diverse content areas, utilizing a large variety of dependent variables and many different types of statistical tests’ (p. 146). That is, the standardization was necessary for Cohen’s systematic review purposes, but it is in principle ill-fitting for power analysis within the context of any specific testing situation (Baguley, 2009). Cohen’s benchmarks have unfortunately transformed into generic rules of thumb, employed by the applied researcher who has no idea of what effect size they are actually interested in, or knows not how to conceive of it. Cohen has even mentioned regretting the fact that he created them in the first place (Funder & Ozer, 2019). Ideally, an effect size is devised in terms of an unstandardized effect and the variance of the measurements, and standardized alternatives should only be employed if the former is untenable–and one should then argue why that is the case (Vankelecom et al., 2024). It is sensible to choose the smallest effect size that is of interest (SESOI), thus guaranteeing statistical power for the ‘worst case scenario’, but also optimizing the study to that effect (for extensive details, see Giner-Sorolla et al., 2024). The relatively smaller variability of sample sizes for studies with power calculations as compared to studies without may indicate that research which includes a report of a power analysis indeed leads to less resource waste, both in the sense that samples are not too small but also not unnecessarily large. However, it may also merely reflect the prominent use of ill-fitting effect size benchmarks, such as d = 0.4 (a ‘medium’ effect), such that sample sizes tend to cluster together accordingly. Nevertheless, our findings suggest that reliance on such benchmarks may be declining, as sample sizes determined by *a priori* power analyses show greater variability in 2020–2021 compared to 2015–2016.

Furthermore, applying rules of thumb instead of careful and precise prior consideration may lead statistical power analysis to become a procedural step rather than a tool for inference. In economics, Goodhart’s law states that when measures become targets, they will no longer be a good measure (Strathern, 1997), and the same broadly applies here: statistical significance was a decision criterion, a measure with a distinct purpose, but has transformed into a target to be chased to achieve other ends, namely publication. Similarly, the whole functionality of statistical power analysis as a tool in statistical inference is at stake when carried out unthinkingly. For example, from the systematic review, it appears that a substantial amount of power analyses employed an effect size of interest based on previous literature, but as already discussed, previous literature likely overestimates effect sizes, and so one paradoxically risks perpetuating the problem of low power and inflated false positive rates by recycling upwardly biased effect size measures and sustaining a vicious cycle fueled by publication bias (see also Nakagawa et al., 2024). Of course, based on the current findings alone, one should not overly generalize to other journals and subfields, as these comments are tentative. Nonetheless, one cannot attempt to fix issues related to statistical power by focusing solely on statistical power, for the concerning problems are multifaceted and interwoven with other issues in the research-publication cycle. Especially, reform efforts should avoid the mere demand for power analyses, and instead incentivize thought-through applications of it (e.g. Giner-Sorolla et al., 2024). Otherwise, the possibility exists that statistical power analysis will become just another bureaucratic hurdle, a box to be ticked if one wishes to publish; part of a new moral economy of what ‘good science’ must *look like* (Penders, 2022), not what it must *be*. The consequences will be higher false positive rates, less or sustained low replicability, ongoing resource waste and the indefinite continuation of psychology’s state of crisis.

### Alternatives to classical power analysis

The results of our systematic review are promising and may be indicative of a positive evolution. However, one must acknowledge that the estimated overall prevalence of power analysis reporting is still low in the journals being considered (only 30% overall in 2020–2021). One reason for this persistent low prevalence might be that, despite the increasing amount of power tutorials, performing an *a priori* power analysis remains challenging (Abraham & Russell, 2008). For example, the population effect size and the nuisance parameters (e.g. the variance of the outcome) of a study are typically not known at the study design phase, yet they are needed to calculate the sample size that is required to detect the population effect with a sufficiently powered research procedure. Powering the experimental design for a SESOI instead of for the anticipated population effect might already partially mitigate this problem; however, the challenge of estimating the nuisance parameters *a priori* remains. Failing to obtain reliable prior estimates of these nuisance parameters will result in incorrect sample size estimation and will therefore render the power analysis untrustworthy. Moreover, due to the complexity of the experimental design that is often employed, researchers may tend to refrain from performing exact power calculations for the effect of interest because these calculations may become increasingly analytically tedious or even impossible (e.g., in settings requiring computationally intensive statistical methods). Researchers may instead prefer omnibus measures in their power calculations, potentially resulting in an insufficient sample size for testing the effect of interest.

Adaptive designs, which are well known and employed within the field of clinical trials, may offer a solution in some cases (Wassmer & Brannath, 2016). Adaptive designs contradict fixed sample designs in that they allow various features of the study design (such as the sample size) to be modified during the study. One such design that might help overcome the abovementioned challenge regarding the unknown nuisance parameters is the *internal pilot study* design (Birkett & Day, 1994; Wittes & Brittain, 1990). In this design, the first batch of collected data is used to derive an estimate of the study’s nuisance parameters, which are in turn used to re-estimate the final sample size. The decisive power analysis is therefore not performed ‘*a priori*’ but rather ‘mid-study’ where the nuisance parameters (and only these parameters) are estimated based on the first collected data. Importantly, statistical inference should be drawn only after all data are gathered. If only a single statistical test is conducted at the end of data collection, the type I error rate will stay approximately at the nominal level, provided the initial ‘internal pilot’ batch is sufficiently large. This design can be applied for different types of statistical tests, however, for a tutorial and in-depth review of this design for the independent samples t-test, see Vankelecom et al. (2024).

Another type of adaptive design, which is a suitable alternative to *a priori* power analysis, is *information-based monitoring* (Tsiatis, 2006). Here, the precision of the estimator of the parameter of interest is continuously monitored during the study, and data collection only stops when the estimator’s precision is large enough (or equivalently, when this estimator’s variance is sufficiently small) to detect a prespecified SESOI. It can be shown that reaching this threshold results in a test with the desired level of power and type I error rate (Friede & Miller, 2012). While the disadvantage of this method is that the required sample size is in principle unknown at the start of the study, it bypasses the difficulty of specifying the nuisance parameters beforehand and does not need an analytical expression in order to perform the sample size calculation (which is a requirement for the internal pilot study design), making the information-based monitoring approach particularly useful for studies with complex statistical analyses.

### Limitations and future research

Despite the scope of the current systematic review, several limitations should be highlighted. First, all analyzed journals were published by the American Psychological Association (APA). While the process of randomly selecting the journals was aimed at increasing comparability and internal validity, we acknowledge that considering only APA-listed journals limits generalizability. For instance, the APA task force recently introduced (non-binding) journal article reporting standards that apply to all APA-listed journals (JARS; Appelbaum et al., 2018). Our results have shown that several of the included journals have effectively implemented these standards in the submission guidelines. While implementing these practices is a positive evolution, future research could be aimed at evaluating whether similar results can be found for articles by other publishers in psychology.

A second limitation pertains to the fact that we did not keep track of the number of analyses that were conducted within each article. Although we did collect information on all experiments that used different samples, we did not look into the number of tests performed for each of these experiments within articles. Maintaining a record of the number of statistical tests could provide additional insights into the perceived justifiability of performing tests based on a single power analysis. Future research could also collect information on the specific software and method used to perform the power analyses (e.g., G*Power [Faul et al., 2007], analytic formula, simulation, etc).

Third, our review could not detect a reported *a priori* sample size calculation that was performed after having analyzed the data, using the obtained ES (i.e., a *post hoc* analysis in reality). Reporting a power analysis as *a priori* is only better scientific practice than reporting nothing when the analysis is actually conducted before the data were obtained. If researchers falsely report an analysis as an *a priori* calculation whilst having used an observed ES, our measure clearly fails its purpose.

Fourth, since we collected information on the different experiments within an article, we also had to decide on how to retain a single experiment. In doing so, we always chose the experiment that did conduct a power analysis (if there was one) and that had the highest sample size. Hence, the results of our review depict a ‘best case scenario’ of the true situation.

Finally, the lack of uniformity in power analysis reporting practices complicated the encoding of the articles (e.g., deciding on whether a power analysis constitutes a true *a priori* versus sensitivity analysis was not always unambiguous). Although we minimized the risk of incorrectly coding these articles by discussing all ambiguous cases, there is still some inherent subjectivity involved. One should therefore critically interpret this distinction between *a priori* power analysis and sensitivity analysis.

## Conclusion

We argue in this paper how insufficient statistical power is one of the key issues that sustains the purported replication crisis, and how this leads to an increase in false discovery rate and an overestimation of effect sizes. As a result of publication bias, the effect sizes reported in the literature to base *a priori* power analysis on are already flawed, which risks perpetuating a cycle of underpowered studies and overestimated effect sizes. We present descriptive results of a systematic review encompassing 903 empirical research articles published in 24 APA-listed journals. In line with previous work (Fraley et al., 2022), we conclude that the practice of power analysis reporting has increased, showing a promising evolution, from 10% of the 453 sampled studies in 2015 and 2016 to 30% of the 450 sampled articles published in 2020 and 2021. This increase sheds new light on the replication crisis and demonstrates that psychological science is slowly evolving towards a more credible scientific field (see also Nosek et al., 2022); that is, insofar as mere reporting of having conducted a power analysis goes. We present discipline- and journal impact factor quartile-specific results and find that social psychology and experimental psychology journals, as well as Q1 and Q2 journals, have made the strongest progress in reporting a power analysis. A promising finding in this regard is that several included journals have imposed sample size justification criteria since 2016, which provides an intuitive explanation for the relatively steep increase in power analysis reporting. However, we argue that both reporting standards and the justification regarding the many choices that must be made prior to conducting a power analysis have to improve.

## Data Accessibility Statement

The dataset and the code to reproduce the results of the systematic review, as well as the supplementary materials, are made available on our OSF-repository: https://osf.io/fhtua/.

## Footnotes

We would like to note that in recent years, the name *replication crisis* has become controversial itself. Specifically, authors have called into question whether a failure to replicate is itself necessarily problematic from a meta-scientific point of view, and have argued, to the degree that it constitutes a misnomer, that reform efforts in psychology are currently based on conceptual ambiguities surrounding replication proper (e.g., Amrhein et al., 2019; Buzbas et al., 2023). The current paper sticks to the classic nomenclature for simplicity’s sake, though the authors acknowledge the putative nature of the crisis as one concerning *replication* itself and not, e.g., *theory*.

A non-exhaustive overview of social science studies that use registered reports can be found on the following Zotero collection: https://www.zotero.org/groups/479248/osf/collections/KEJP68G9.

https://www.apa.org/pubs/journals/resources/find-journal.

We categorized power analyses that were performed with a theoretical effect size as either *a priori* power analysis (when solving for the required sample size) or sensitivity analysis (when solving for the effect size or power given a particular sample size), and power analyses that were performed with the obtained effect size from the study as *post hoc* power analysis (O’Keefe, 2007). For that reason, the power analyses with minimal detectable effect sizes that were described by the authors as *post hoc* (because they were performed *after* the study to check the obtained power with the collected sample) were categorized by us as sensitivity analyses.

Several journals had different JIF quartiles in 2015–2016 as compared to 2020–2021. The quartile that is shown on the plot corresponds to the quartile with respect to that specific year for which the article was analyzed.

39 articles performed a sensitivity analysis where the attained power for a fixed sample size and fixed effect size of interest was calculated. These articles are therefore not included in these numbers.

15 articles performed a sensitivity analysis where the detectable effect size for a fixed sample size and fixed desired power level was deducted. These articles are therefore not included in these numbers.

2 articles with RMSEA as effect size (values of 0.05 and 0.08), 1 article with OR as effect size (value of 1.8), and the 38 articles that did not mention the type of ES were excluded from this boxplot.

## References

[1] Abraham, W. T., & Russell, D. W. (2008). Statistical power analysis in psychological research. Social and Personality Psychology Compass, 2(1), 283–301. 10.1111/j.1751-9004.2007.00052.x

[2] Agnoli, F., Wicherts, J. M., Veldkamp, C. L., Albiero, P., & Cubelli, R. (2017). Questionable research practices among Italian research psychologists. PloS one, 12(3). 10.1371/journal.pone.0172792 PMC5351839 28296929

[3] Amrhein, V., Trafimow, D., & Greenland, S. (2019). Inferential statistics as descriptive statistics: There is no replication crisis if we don’t expect replication. The American Statistician, 73(sup1), 262–270. 10.1080/00031305.2018.1543137

[4] Appelbaum, M. I., Cooper, H., Kline, R. B., Mayo-Wilson, E., Nezu, A. M., & Rao, S. M. (2018). Journal article reporting standards for quantitative research in psychology: The APA Publications and Communications Board task force report. American Psychologist, 73(1), 3–25. 10.1037/amp0000191 29345484

[5] Asendorpf, J. B., Conner, M., De Fruyt, F., De Houwer, J., Denissen, J. J. A., Fiedler, K., Fiedler, S., Fun-der, D. C., Kliegl, R., Nosek, B. A., Perugini, M., Roberts, B. W., Schmitt, M., Vanaken, M. A. G., Weber, H., & Wicherts, J. M. (2013). Recommendations for increasing replicability in psychology. European Journal of Personality, 27(2), 108–119. 10.1002/per.1919

[6] Baguley, T. (2009). Standardized or simple effect size: What should be reported? British Journal Of Psychology, 100(3), 603–617. 10.1348/000712608x377117 19017432

[7] Birkett, M. A., & Day, S. J. (1994). Internal pilot studies for estimating sample size. Statistics in Medicine, 13(23–24), 2455–2463. 10.1002/sim.4780132309 7701146

[8] Bishop, D. (2019). Rein in the four horsemen of irreproducibility. Nature, 568(7753), 435. 10.1038/d41586-019-01307-2 31019328

[9] Brodeur, A., Cook, N., Hartley, J., & Heyes, A. (2022). Do pre-registration and pre-analysis plans reduce p-hacking and publication bias? 10.2139/ssrn.4180594

[10] Brysbaert, M. (2019). How Many Participants Do We Have to Include in Properly Powered Experiments? A Tutorial of Power Analysis with Reference Tables. Journal of cognition, 2(1), 16. 10.5334/joc.72 31517234 PMC6640316

[11] Button, K. S., Ioannidis, J. P. A., Mokrysz, C., Nosek, B. A., Flint, J., Robinson, E., & Munafò, M. R. (2013). Power failure: why small sample size undermines the reliability of neuroscience. Nature Reviews Neuroscience, 14(5), 365–376. 10.1038/nrn3475 23571845

[12] Buzbas, E. O., Devezer, B., & Baumgaertner, B. (2023). The logical structure of experiments lays the foundation for a theory of reproducibility. Royal Society Open Science, 10, Article 221042. 10.1098/rsos.221042 36938532 PMC10014247

[13] Cohen, J. (1962). The statistical power of abnormal-social psychological research: A review. The Journal of Abnormal and Social Psychology, 65(3), 145–153. 10.1037/h0045186 13880271

[14] Cohen, J. (1988). Statistical Power Analysis for the Behavioral Sciences (2nd ed.). Routledge. 10.4324/9780203771587

[15] Cohen, J. (1992). A power primer. Psychological Bulletin, 112(1), 155–159. 10.1037/0033-2909.112.1.155 19565683

[16] Colling, L. J., & Szűcs, D. (2021). Statistical inference and the replication crisis. Review of Philosophy and Psychology, 12(1), 121–147. 10.1007/s13164-018-0421-4

[17] Cumming, G. (2008). Replication and p Intervals: p Values Predict the Future Only Vaguely, but Confidence Intervals Do Much Better. Perspectives on Psychological Science, 3(4), 286–300. 10.1111/j.1745-6924.2008.00079.x 26158948

[18] Cumming, G. (2012). Understanding the new statistics: Effect sizes, confidence intervals, and meta-analysis. Routledge. 10.4324/9780203807002

[19] Davis, S., Johnson, A. H., Lynch, T., Gray, L., Pryor, E. R., Azuero, A., Soistmann, H. C., Phillips, S. R., & Rice, M. (2020). Inclusion of Effect Size Measures and Clinical Relevance in Research Papers. Nursing Research, 70(3), 222–230. 10.1097/nnr.0000000000000494 PMC8939369 33323832

[20] De Rond, M., & Miller, A. N. (2005). Publish or perish: Bane or boon of academic life? Journal of management inquiry, 14(4), 321–329. 10.1177/1056492605276850

[21] Doyen, S., Klein, O., Pichon, C. L., & Cleeremans, A. (2012). Behavioral priming: it’s all in the mind, but whose mind? PloS one, 7(1). 10.1371/journal.pone.0029081 PMC3261136 22279526

[22] Ebersole, C. R., Atherton, O. E., Belanger, A. L., Skulborstad, H. M., Allen, J., Banks, J., Baranski, E., Bernstein, M. J., Bonfiglio, D. B. V., Boucher, L., Brown, E. R., Budiman, N. I., Cairo, A. H., Capaldi, C. A., Chartier, C. R., Chung, J. M., Cicero, D. C., Coleman, J. A., Conway, J., … Nosek, B. A. (2016). Many Labs 3: Evaluating participant pool quality across the academic semester via replication. Journal Of Experimental Social Psychology, 67, 68–82. 10.1016/j.jesp.2015.10.012

[23] Ebersole, C. R., Mathur, M. B., Baranski, E., Bart-Plange, D.-J., Buttrick, N. R., Chartier, C. R., Corker, K. S., Corley, M., Hartshorne, J. K., IJzerman, H., Lazarević, L. B., Rabagliati, H., Ropovik, I., Aczel, B., Aeschbach, L. F., Andrighetto, L., Arnal, J. D., Arrow, H., Babincak, P., … Nosek, B. A. (2020). Many Labs 5: Testing pre-data-collection peer review as an intervention to increase replicability. Advances in Methods and Practices in Psychological Science, 3(3), 309–331. 10.1177/2515245920958687

[24] Ehde, D. M. (2018). Opening editorial: Rehabilitation Psychology [Editorial]. Rehabilitation Psychology, 63(2), 167–169. 10.1037/rep0000233 29878824

[25] Fanelli, D. (2012). Negative results are disappearing from most disciplines and countries. Scientometrics, 90(3), 891–904. 10.1007/s11192-011-0494-7

[26] Faul, F., Erdfelder, E., Lang, A. G., & Buchner, A. (2007). G*Power 3: A flexible statistical power analysis program for the social, behavioral, and biomedical sciences. Behavior Research Methods, 39(2), 175–191. 10.3758/bf03193146 17695343

[27] Ferguson, C. J., & Brannick, M. T. (2012). Publication bias in psychological science: Prevalence, methods for identifying and controlling, and implications for the use of meta-analyses. Psychological Methods, 17(1), 120–128. 10.1037/a0024445 21787082

[28] Fraley, R. C., Chong, J. Y., Baacke, K. A., Greco, A. J., Guan, H., & Vazire, S. (2022). Journal N-pact factors from 2011 to 2019: evaluating the quality of social/personality journals with respect to sample size and statistical power. Advances in Methods and Practices in Psychological Science, 5(4). 10.1177/25152459231175075

[29] Fraley, R. C., & Vazire, S. (2014). The N-Pact Factor: Evaluating the Quality of Empirical Journals with Respect to Sample Size and Statistical Power. PLOS ONE, 9(10). 10.1371/journal.pone.0109019 PMC4189949 25296159

[30] Francis, G. (2012). Publication bias and the failure of replication in experimental psychology. Psychonomic Bulletin & Review, 19, 975–991. 10.3758/s13423-012-0322-y 23055145

[31] Fraser, H., Parker, T., Nakagawa, S., Barnett, A., & Fidler, F. (2018). Questionable research practices in ecology and evolution. PLOS ONE, 13(7), e0200303. 10.1371/journal.pone.0200303 30011289 PMC6047784

[32] Friede, T., & Miller, F. (2012). Blinded continuous monitoring of nuisance parameters in clinical trials. Journal of the Royal Statistical Society Series C (Applied Statistics), 61(4), 601–618. 10.1111/j.1467-9876.2011.01029.x

[33] Friese, M., & Frankenbach, J. (2020). p-Hacking and publication bias interact to distort meta-analytic effect size estimates. Psychological Methods, 25(4), 456–471. 10.1037/met0000246 31789538

[34] Fritz, A., Scherndl, T., & Kühberger, A. (2012). A comprehensive review of reporting practices in psychological journals: Are effect sizes really enough? Theory & Psychology, 23(1), 98–122. 10.1177/0959354312436870

[35] Funder, D. C., & Ozer, D. J. (2019). Evaluating effect size in psychological research: Sense and nonsense. Advances in Methods and Practices in Psychological Science, 2(2), 156–168. 10.1177/2515245919847202

[36] Gauthier, I. (2018). Inaugural editorial [Editorial]. Journal of Experimental Psychology: Human Perception and Performance, 44(1), 1. 10.1037/xhp0000519 29309191

[37] Giner-Sorolla, R. (2018). From crisis of evidence to a “crisis” of relevance? incentive-based answers for Social Psychology’s perennial relevance worries. European Review of Social Psychology, 30(1), 1–38. 10.1080/10463283.2018.1542902

[38] Giner-Sorolla, R., Montoya, A. K., Reifman, A., Carpenter, T., Lewis, N. A., Jr., Aberson, C. L., Bostyn, D. H., Conrique, B. G., Ng, B. W., Schoemann, A. M., & Soderberg, C. (2024). Power to detect what? Considerations for planning and evaluating sample size. Personality and Social Psychology Review, 28(3), 276–301. 10.1177/10888683241228328 38345247 PMC11193916

[39] Greenwald, A. G. (1975). Consequences of prejudice against the null hypothesis. Psychological bulletin, 82(1), 1–20. 10.1037/h0076157

[40] Head, M. L., Holman, L., Lanfear, R., Kahn, A. T., & Jennions, M. D. (2015). The extent and consequences of p-hacking in science. PLoS biology, 13(3), e1002106. 10.1371/journal.pbio.1002106 25768323 PMC4359000

[41] Hoenig, J. M., & Heisey, D. M. (2001). The Abuse of Power. The American Statistician, 55(1), 19–24. 10.1198/000313001300339897

[42] Ioannidis, J. P. A. (2005). Why most published research findings are false. PLoS ONE, 2(8), e124. 10.1371/journal.pmed.0020124 PMC1182327 16060722

[43] Ioannidis, J. P. A. (2008). Why most discovered true associations are inflated. Epidemiology, 19(5), 640–648. 10.1097/ede.0b013e31818131e7 18633328

[44] Ioannidis, J. P. A., Stanley, T. D., & Doucouliagos, H. (2017). The Power of Bias in Economics Research. The Economic Journal, 127(605), F236–F265. 10.1111/ecoj.12461

[45] John, L. K., Loewenstein, G., & Prelec, D. (2012). Measuring the Prevalence of Questionable Research Practices With Incentives for Truth Telling. Psychological Science, 23(5), 524–532. 10.1177/0956797611430953 22508865

[46] Kitayama, S. (2017). Journal of Personality and Social Psychology: Attitudes and social cognition [Editorial]. Journal of Personality and Social Psychology, 112(3), 357–360. 10.1037/pspa0000077 28221091

[47] Klein, R. A., Cook, C. L., Ebersole, C. R., Vitiello, C., Nosek, B. A., Hilgard, J., Ahn, P. H., Brady, A. J., Chartier, C. R., Christopherson, C. D., Clay, S., Collisson, B., Crawford, J. T., Cromar, R., Gardiner, G., Gosnell, C. L., Grahe, J., Hall, C., Howard, I., … Ratliff, K. A. (2022). Many labs 4: Failure to replicate mortality salience effect with and without original author involvement. Collabra: Psychology, 8(1), 1–15. 10.1525/collabra.35271

[48] Klein, R. A., Ratliff, K., Vianello, M., Adams, A. B., Jr., Bahník, S., Bernstein, N. B., … Nosek, B. A. (2014). Investigating variation in replicability: A “Many Labs” Replication Project. Social Psychology, 45, 142–152. 10.1027/1864-9335/a000178

[49] Klein, R. A., Vianello, M., Hasselman, F., Adams, B. G., Adams, R. B., Jr., Alper, S., … Sowden, W. (2018). Many Labs 2: Investigating variation in replicability across samples and settings. Advances in Methods and Practices in Psychological Science, 1(4), 443–490. 10.1177/2515245918810225

[50] Lakens, D. (2013). Calculating and reporting effect sizes to facilitate cumulative science: a practical primer for t-tests and ANOVAs. Frontiers in psychology, 4, 863. 10.3389/fpsyg.2013.00863 24324449 PMC3840331

[51] Lakens, D. (2022). Sample Size Justification. Collabra: Psychology, 8(1). 10.1525/collabra.33267

[52] Lakens, D., Adolfi, F., Albers, C. J., Anvari, F., Apps, M. A. J., Argamon, S., Baguley, T., Becker, R., Benning, S. D., Bradford, D. E., Buchanan, E. M., Caldwell, A. R., Van Calster, B., Carlsson, R., Chen, S., Chung, B., Colling, L., Collins, G. S., Crook, Z., … Zwaan, R. A. (2018). Justify your alpha. Nature Human Behaviour, 2(3), 168–171. 10.1038/s41562-018-0311-x

[53] Levitt, H. M., Bamberg, M., Creswell, J. W., Frost, D. M., Josselson, R., & Suárez-Orozco, C. (2018). Journal article reporting standards for qualitative primary, qualitative meta-analytic, and mixed methods research in psychology: The APA Publications and Communications Board task force report. American Psychologist, 73(1), 26–46. 10.1037/amp0000151 29345485

[54] Linder, C., & Farahbakhsh, S. (2020). Unfolding the black box of questionable research practices: Where is the line between acceptable and unacceptable practices? Business Ethics Quarterly, 30(3), 335–360. 10.1017/beq.2019.52

[55] Lindstromberg, S. (2023). The winner’s curse and related perils of low statistical power – spelled out and illustrated. Research Methods in Applied Linguistics, 2(3). 10.1016/j.rmal.2023.100059

[56] Maxwell, S. E. (2004). The persistence of underpowered studies in psychological research: Causes, consequences, and remedies. Psychological Methods, 9(2), 147–163. 10.1037/1082-989X.9.2.147 15137886

[57] Morawski, J. G. (2019). The replication crisis: How might philosophy and theory of psychology be of use? Journal Of Theoretical And Philosophical Psychology, 39(4), 218–238. 10.1037/teo0000129

[58] Moussa, S., & Charlton, A. (2023). Retraction (mal)practices of elite marketing and social psychology journals in the Dirk Smeesters’ research misconduct case. Accountability in Research, 1–16. 10.1080/08989621.2022.2164489 36631998

[59] Munafò, M. R., Nosek, B. A., Bishop, D. V. M., Button, K. S., Chambers, C. D., Du Sert, N. P., Simonsohn, U., Wagenmakers, E. J., Ware, J., & Ioannidis, J. P. A. (2017). A manifesto for reproducible science. Nature Human Behaviour, 1(1). 10.1038/s41562-016-0021 PMC7610724 33954258

[60] Nakagawa, S., Lagisz, M., Yang, Y., & Drobniak, S. M. (2024). Finding the right power balance: Better study design and collaboration can reduce dependence on statistical power. PLOS Biology, 22(1), e3002423. 10.1371/journal.pbio.3002423 38190355 PMC10773938

[61] Nosek, B. A., Alter, G., Banks, G. C., Borsboom, D., Bowman, S., Breckler, S. J., Buck, S., Chambers, C., Chin, G., Christensen, G., Contestabile, M., Dafoe, A., Eich, E., Freese, J., Glennerster, R., Goroff, D. L., Green, D. P., Hesse, B. W., Humphreys, M., … Yarkoni, T. (2015). Promoting an open research culture. Science, 348(6242), 1422–1425. 10.1126/science.aab2374 26113702 PMC4550299

[62] Nosek, B. A., Ebersole, C. R., DeHaven, A. C., & Mellor, D. T. (2018). The preregistration revolution. Proceedings Of The National Academy Of Sciences Of The United States Of America, 115(11), 2600–2606. 10.1073/pnas.1708274114 29531091 PMC5856500

[63] Nosek, B. A., Hardwicke, T. E., Moshontz, H., Allard, A., Corker, K. S., Dreber, A., Fidler, F., Hilgard, J., Struhl, M. K., Nuijten, M. B., Rohrer, J. M., Romero, F., Scheel, A. M., Scherer, L. D., Schönbrodt, F. D., & Vazire, S. (2022). Replicability, Robustness, and Reproducibility in Psychological Science. Annual Review Of Psychology, 73(1), 719–748. 10.1146/annurev-psych-020821-114157 34665669

[64] Nosek, B. A., & Lakens, D. (2014). Registered reports. Social Psychology, 45(3), 137–141. 10.1027/1864-9335/a000192

[65] Nosek, B. A., Spies, J. R., & Motyl, M. (2012). Scientific utopia. Perspectives On Psychological Science, 7(6), 615–631. 10.1177/1745691612459058 26168121 PMC10540222

[66] O’Keefe, D. J. (2007). Brief report: Post hoc power, observed power, a priori power, retrospective power, prospective power, achieved power: Sorting out appropriate uses of statistical power analyses. Communication Methods and Measures, 1(4), 291–299. 10.1080/19312450701641375

[67] Open Science Collaboration. (2015). Estimating the reproducibility of psychological science. Science, 349(6251). 10.1126/science.aac4716 26315443

[68] Pashler, H., & Harris, C. R. (2012). Is the Replicability Crisis Overblown? Three Arguments Examined. Perspectives On Psychological Science, 7(6), 531–536. 10.1177/1745691612463401 26168109

[69] Pashler, H., & Wagenmakers, E. (2012). Editors’ Introduction to the Special Section on Replicability in Psychological Science. Perspectives On Psychological Science, 7(6), 528–530. 10.1177/1745691612465253 26168108

[70] Pek, J., Hoisington-Shaw, K. J., & Wegener, D. (2024). Uses of uncertain statistical power: Designing future studies, not evaluating completed studies. Psychological Methods. Advance online publication. 10.1037/met0000577 39298193

[71] Penders, B. (2022). Process and Bureaucracy: Scientific Reform as Civilisation. Bulletin Of Science, Technology & Society, 42(4), 107–116. 10.1177/02704676221126388

[72] Perugini, M., Gallucci, M., & Costantini, G. (2018). A practical primer to power analysis for simple experimental designs. Revue Internationale de Psychologie Sociale, 31(1), 1–23. 10.5334/irsp.181

[73] Pupovac, V., Prijić-Samaržija, S., & Petrovečki, M. (2017). Research misconduct in the Croatian scientific community: a survey assessing the forms and characteristics of research misconduct. Science and engineering ethics, 23, 165–181. 10.1007/s11948-016-9767-0 26940319

[74] Rodgers, J. L., & Shrout, P. E. (2018). Psychology’s replication crisis as scientific opportunity: A précis for policymakers. Policy Insights from the Behavioral and Brain Sciences, 5(1), 134–141. 10.1177/2372732217749254

[75] Rosenthal, R. (1979). The “file drawer problem” and tolerance for null results. Psychological Bulletin, 86(3), 638–641. 10.1037/0033-2909.86.3.638

[76] Schauer, J. M., & Hedges, L. V. (2020). Assessing heterogeneity and power in replications of psychological experiments. Psychological bulletin, 146(8), 701–719. 10.1037/bul0000232 32271029

[77] Sedlmeier, P., & Gigerenzer, G. (1989). Do studies of statistical power have an effect on the power of studies? Psychological Bulletin, 105(2), 309–316. 10.1037/0033-2909.105.2.309

[78] Shrout, P. E., & Rodgers, J. L. (2018). Psychology, science, and knowledge construction: Broadening perspectives from the replication crisis. Annual review of psychology, 69, 487–510. 10.1146/annurev-psych-122216-011845 29300688

[79] Simmons, J. P., Nelson, L. D., & Simonsohn, U. (2011). False-positive psychology: Undisclosed flexibility in data collection and analysis allows presenting anything as significant. Psychological science, 22(11), 1359–1366. 10.1177/0956797611417632 22006061

[80] Soderberg, C. K., Errington, T. M., Schiavone, S. R., Bottesini, J., Thorn, F. S., Vazire, S., … Nosek, B. A. (2021). Initial evidence of research quality of registered reports compared with the standard publishing model. Nature Human Behaviour, 5(8), 990–997. 10.1038/s41562-021-01142-4 34168323

[81] Stanley, T. D., Carter, E. C., & Doucouliagos, H. (2018). What meta-analyses reveal about the replicability of psychological research. Psychological bulletin, 144(12), 1325. 10.1037/bul0000169 30321017

[82] Stanley, T. D., Doucouliagos, H., & Ioannidis, J. P. A. (2021). Retrospective median power, false positive meta-analysis and large-scale replication. Research Synthesis Methods, 13(1), 88–108. 10.1002/jrsm.1529 34628722

[83] Stefan, A. M., & Schönbrodt, F. D. (2023). Big little lies: A compendium and simulation of p-hacking strategies. Royal Society Open Science, 10(2), 220346. 10.1098/rsos.220346 36778954 PMC9905987

[84] Strathern, M. (1997). ‘Improving ratings’: audit in the British University system. European Review, 5(3), 305–321. 10.1002/(SICI)1234-981X(199707)5:3<305::AID-EURO184>3.0.CO;2-4

[85] Stroebe, W., & Strack, F. (2014). The Alleged Crisis and the Illusion of Exact Replication. Perspectives On Psychological Science, 9(1), 59–71. 10.1177/1745691613514450 26173241

[86] Świątkowski, W., & Dompnier, B. (2017). Replicability Crisis in Social Psychology: Looking at the Past to Find New Pathways for the Future. International Review Of Social Psychology, 30(1), 111–124. 10.5334/irsp.66

[87] Swift, J. K., Christopherson, C. D., Bird, M. O., Zöld, A., & Goode, J. (2022). Questionable research practices among faculty and students in APA-accredited clinical and counseling psychology doctoral programs. Training and Education in Professional Psychology, 16(3), 299–305. 10.1037/tep0000322

[88] Szucs, D., & Ioannidis, J. P. (2017). Empirical assessment of published effect sizes and power in the recent cognitive neuroscience and psychology literature. PLoS biology, 15(3), e2000797. 10.1371/journal.pbio.2000797 28253258 PMC5333800

[89] Tressoldi, P. E., & Giofré, D. (2015). The pervasive avoidance of prospective statistical power: Major consequences and practical solutions. Frontiers in Psychology, 6, 137497. 10.3389/fpsyg.2015.00726 PMC4446541 26074863

[90] Tsiatis, A. A. (2006). Information-based monitoring of clinical trials. Statistics in Medicine, 25(19), 3236–3244. 10.1002/sim.2625 16927248

[91] Van Zwet, E. W., & Cator, E. (2021). The significance filter, the winner’s curse and the need to shrink. Statistica Neerlandica, 75(4), 437–452. 10.1111/stan.12241

[92] Vankelecom, L., Loeys, T., & Moerkerke, B. (2024). How to Safely Reassess Variability and Adapt Sample Size? A Primer for the Independent Samples t Test. Advances in Methods And Practices in Psychological Science, 7(1). 10.1177/25152459231212128

[93] Vankov, I., Bowers, J., & Munafò, M. R. (2014). On the persistence of low power in psychological science. Quarterly Journal of Experimental Psychology, 67, 1037–1040. DOI: 10.1080/17470218.2014.885986 PMC4961230 24528377

[94] Wagenmakers, E., Wetzels, R., Borsboom, D., & Van Der Maas, H. L. J. (2011). Why psychologists must change the way they analyze their data: The case of psi: Comment on Bem (2011). Journal Of Personality And Social Psychology, 100(3), 426–432. 10.1037/a0022790 21280965

[95] Wang, Y. A. (2023). How to Conduct Power Analysis for Structural Equation Models: A Practical Primer. PsyArXiv. 10.31234/osf.io/4n3uk

[96] Wassmer, G., & Brannath, W. (2016). Group sequential and confirmatory adaptive designs in clinical trials. Springer. 10.1007/978-3-319-32562-0

[97] Wicherts, J. (2011). Psychology must learn a lesson from fraud case. Nature, 480, 7. 10.1038/480007a 22129686

[98] Wicherts, J. M., Veldkamp, C. L., Augusteijn, H. E., Bakker, M., Van Aert, R., & Van Assen, M. A. (2016). Degrees of freedom in planning, running, analyzing, and reporting psychological studies: A checklist to avoid p-hacking. Frontiers in psychology, 7. 10.3389/fpsyg.2016.01832 PMC5122713 27933012

[99] Wiggins, B. J., & Christopherson, C. D. (2019). The replication crisis in psychology: An overview for theoretical and philosophical psychology. Journal Of Theoretical And Philosophical Psychology, 39(4), 202–217. 10.1037/teo0000137

[100] Wittes, J., & Brittain, E. (1990). The role of internal pilot studies in increasing the efficiency of clinical trials. Statistics in Medicine, 9(1–2), 65–71. 10.1002/sim.4780090110 2345839

